# Improving access to early intervention for autism – findings from a proof-of-principle cascaded task-sharing naturalistic developmental behavioural intervention in South Africa

**DOI:** 10.21203/rs.3.rs-2624968/v1

**Published:** 2023-03-01

**Authors:** Amber D Rieder, Marisa Viljoen, Noleen Seris, Nokuthula Shabalala, Minkateko Ndlovu, Elizabeth L Turner, Ryan Simmons, Petrus J Vries, Lauren Franz

**Affiliations:** Centre for Autism Research in Africa, Division of Child and Adolescent Psychiatry, University of Cape Town; Centre for Autism Research in Africa, Division of Child and Adolescent Psychiatry, University of Cape Town; Centre for Autism Research in Africa, Division of Child and Adolescent Psychiatry, University of Cape Town; Centre for Autism Research in Africa, Division of Child and Adolescent Psychiatry, University of Cape Town; Centre for Autism Research in Africa, Division of Child and Adolescent Psychiatry, University of Cape Town; Department of Biostatistics and Bioinformatics, Duke University; Department of Biostatistics and Bioinformatics, Duke University; Centre for Autism Research in Africa, Division of Child and Adolescent Psychiatry, University of Cape Town; Duke Center for Autism and Brain Development, Department of Psychiatry and Behavioral Sciences, Duke University

**Keywords:** Autism, early intervention, task-sharing, naturalistic developmental behavioural intervention, proof-of-principle study

## Abstract

**Background:**

Despite the high number of children living with neurodevelopmental disabilities in sub–Saharan Africa, access to early intervention is almost non-existent. It is therefore important to develop feasible, scalable early autism intervention that can be integrated into systems of care. While Naturalistic Developmental Behavioural Intervention (NDBI) has emerged as an evidence-based intervention approach, implementation gaps exist globally, and task-sharing approaches may address access gaps. In this South African proof-of-principle pilot study, we set out to answer two questions about a 12-session cascaded task-sharing NDBI – whether the approach could be delivered with fidelity, and whether we could identify signals of change in child and caregiver outcomes.

**Methods:**

We utilized a single-arm pre-post design. Fidelity (non-specialists, caregivers), caregiver outcomes (stress, sense of competence), and child outcomes (developmental, adaptive) were measured at baseline (T1) and follow-up (T2). Ten caregiver-child dyads and four non-specialists participated. Pre-to-post summary statistics were presented alongside individual trajectories. Non-parametric Wilcoxon signed rank test for paired samples was used to compare group medians between T1 and T2.

**Results:**

Caregiver implementation fidelity increased in 10/10 participants. Non-specialists demonstrated a significant increase in coaching fidelity (increases in 7/10 dyads). Significant gains were seen on two Griffiths-III subscales (Language/Communication – 9/10 improved, Foundations of Learning – 10/10 improved) and on the General Developmental Quotient (9/10 improved). Significant gains were also seen on two Vineland Adaptive Behaviour Scales (Third Edition) subscales (Communication – 9/10 improved, Socialization −6/10 improved) and in the Adaptive Behaviour Standard Score (9/10 improved). Caregiver sense of competence improved in 7/10 caregivers and caregiver stress in 6/10 caregivers.

**Conclusions:**

This proof-of-principle pilot study of the first cascaded task-sharing NDBI in Sub-Saharan Africa provided fidelity and intervention outcome data which supported the potential of such approaches in low-resource contexts. Larger studies are needed to expand on the evidence-base and answer questions on intervention effectiveness and implementation outcomes.

## Background

Lack of services and supports for autistic individuals has been recognized by the World Health Organization as a global public health concern [1]. In low-and-middle-income countries (LMIC), where 95% of autistic people live, services and supports are critically scarce [2–5]. The recent *Lancet* Commission on future care in autism proposed the necessary coordination between healthcare and other sectors such as education to address the gap in access, and to promote programmes that can be personalized, taking into account the preferences, needs, and incurred costs (financial and otherwise) of families and their autistic children [6]. Furthermore, the Commission reiterated the importance of access to timely supports services and that no one, regardless of geographic location or resource availability, should wait for extended periods of time to start interventions that could improve child and family quality of life [6]. Early intervention for autism is important because it can support growth in receptive and expressive language, as well as cognitive abilities, social skills, and adaptive behaviours, with positive downstream effects on the developmental cascade [7–8]. Current evidence-based practice in early intervention blend developmental and behavioural approaches and incorporate caregivers in intervention planning and delivery [9–10].

Naturalistic Developmental Behavioural Intervention (NDBI) is a class of interventions delivered by trained therapists with active caregiver involvement, leverage the opportunity to support developmental growth of children at home and in their daily lives. Early childhood is a particularly sensitive stage of human development in which the brain undergoes rapid growth and maturation, offering a critical window for supportive intervention. Interventions that promote social and communication development during this period of rapid growth have been associated with a cascade of positive short- and long-term functional outcomes [11]. The Early Start Denver Model (ESDM) is an NDBI that promotes child social engagement by embedding social learning opportunities in child preferred routines, thereby heightening the reward value of engagement, and increasing child social attention [7]. Importantly, ESDM supports the development of communication abilities in whichever form is appropriate for the individual so that the child over time becomes able to express their needs, preferences and perspectives, and develop skills that enhance their quality of life. Through ESDM coaching, caregivers can be supported in understanding which social activities their autistic child prefers and how to join with their child in those preferred activities.

### Challenges to meet the needs of families that care for young autistic children in sub-Saharan Africa

A high number of individuals with neurodevelopmental disabilities live in sub-Saharan Africa [5]. This is in part due to limited disability-specific services and supports for the growing number of children now surviving the first 5 years of life [5]. The African continent is undergoing an unprecedented demographic shift in child population size and is projected to reach one billion children over the next three decades, an increase in the under-18 population by two-thirds [12]. Considering sub-Saharan Africa embraces the highest proportion of the world’s children, this demographic shift highlights the importance of developing feasible, sustainable, and contextually anchored early interventions for autistic children that can be integrated into existing systems of care.

Meeting the needs of families who care for young autistic children in LMIC is hampered by significant ongoing systemic challenges. Although 95% of the global population of children and adolescents reside in LMIC, only 10% of mental health research - and even less on neurodevelopmental conditions – has been conducted in these settings [3, 4, 6]. Although the principle of ‘task-sharing’ has been widely promoted as a potential solution to meet demand, a lack of trained professionals and a dearth of specialist services has resulted in many families in LMIC with unmet needs. Evidence-based programmes for neurodevelopmental conditions (such as NDBI) that have been developed and evaluated in high-income countries, remain inaccessible due to inadequate resources dedicated to adaptation, implementation and evaluation of contextually adapted materials and procedures. High-quality research designed to evaluate a range of child (e.g. communication, cognitive, and social development) and caregiver (e.g. caregiver wellbeing and sense of parenting competency) outcomes, alongside an implementation evaluation (e.g. examination of fidelity or the degree to which the delivery of an intervention programme utilizes and adheres to the intended materials and procedures) is limited to non-existent in LMIC [13–14]. Promising scalable non-specialist and caregiver-mediated services and supports, adapted for racially, ethnically, and linguistically diverse families in Africa, remains a significant gap to be addressed [6].

### Challenges to meet the needs of families that care for young autistic children in South Africa

Policies that prioritize early childhood development (ECD) are emerging in sub-Saharan African countries, like South Africa [15]. The enactment of policies that advance ECD goals affirms government commitment to the early childhood period, provides a mandate that supports funding for services, and identifies those accountable for providing care. The National Integrated Early Childhood Development Policy prioritizes children with disabilities, including autism, to ensure equitable access to services [15]. This policy recognizes the South African Governments’ “responsibility to ensure a sufficient number of appropriately qualified human resources, including non-specialist early childhood development practitioners and their supervisors” [15].

Although South Africa is an upper-middle-income country, it remains an economy with one of the highest, persistent inequality rates globally [16]. The imbalance in wealth distribution, along with the inequitable adoption and prioritization of anti-poverty policies aimed at advancing social determinants of health, have engendered stark disparities in access to health and education services [17]. In South Africa, these disparities are perpetuated by the legacy of apartheid with systematic exclusion and subsequent lack of intergenerational economic mobility. This has resulted in the needs of the vast majority of individuals, who may benefit from early intervention, going unmet [18, 19, 20].

There are significant socioeconomic barriers that limit access to private health care services for much of the South African population, and racial variation in expressive language abilities at time of diagnosis has been reported in children accessing public health care services in the Western Cape Province of South Africa [21]. In a 2-year retrospective case review of autistic children who attended a tertiary paediatric neurodevelopmental clinic in the Western Cape Province of South Africa, while 42% of White children were non-verbal at diagnosis, 77% of Coloured children (a South African term for mixed-race), and 94% of Black children were non-verbal at presentation [21]. Similarly, a recent study in the Education system documented a higher-than-expected proportion of autistic children from White racial groups and English-speaking families, when compared with Western Cape provincial demographic data. Structural inequalities impact access to diagnostic evaluation, affordable supports and services, and public awareness of neurodevelopmental conditions [19, 20, 22].

### Non-specialist intervention in low-resource settings

While questions around the importance of cumulative intervention intensity are beginning to emerge, the vast majority of evidence-based early autism interventions are intensive and delivered by trained therapists [23–24]. Both the intensity of intervention and reliance on highly trained therapists act as implementation barriers, particularly in low resource communities. The *Lancet* Commission report introduced the concept of a stepped care approach to service delivery as a framework for equitable resource distribution that supports improvement in outcomes for autistic individuals. This type of approach, where the least resource-intensive service such as low-intensity, non-specialist delivered interventions are offered first, may be particularly well suited to LMIC and other low resource settings [6]. In low-resource contexts, innovative solutions involving redistribution of intervention services to both caregivers *and* non-specialist providers (e.g., ECD practitioners) may address the service gap impacting young autistic children and their families [25]. Task-sharing may improve equity in access, and extend healthcare delivery, particularly in under-resourced contexts. This dual redistribution of roles from specialist to non-specialist providers, and from interventionist to caregiver, is responsive to the realities of low resource environments. Task-sharing may be a key implementation strategy that advances accessibility, through principles of sustainability and scalability.

#### Towards cascaded task-sharing to deliver Naturalistic Developmental Behavioural Intervention in sub-Saharan Africa and meaningful measurement of change

Task-sharing in autism intervention promotes active caregiver involvement via caregiver coaching. Caregiver-implemented intervention, where caregivers are coached in strategies to support their child’s social and communication growth during everyday activities, may be utilized to overcome some service access barriers [26]. Increasingly, evidence both from high- and low-income countries support family-centered models of early intervention, which align with the NDBI approach [12]. Given that caregivers play a central role in early intervention, it is critical that the transactional process whereby caregivers can *both* impact intervention outcomes and be impacted by the intervention be recognized [27]. Furthermore, factors that impact adaptive family functioning, caregiver stress and self-efficacy such as poverty, limited social support, and stressful life events are more prevalent in low-resource settings, making these important contextual considerations in caregiver-implemented intervention [28–31].

Task-sharing the coaching role in caregiver-implemented intervention, from highly trained therapist to non-specialist provider, offers a parallel opportunity to increase access to services. In a meta-analysis of non-specialist delivered intervention, only two studies included autistic participants from LMIC [32]. In these two studies ‘non-specialists’ were certified teachers and therapists who provided intervention directly to the child without caregiver involvement. In a study conducted in India, lay health workers under the supervision of specialists delivered 12 coaching sessions of a developmental autism intervention [33]. While this study provided preliminary evidence of the feasibility and effectiveness of a non-specialist delivered early autism intervention in India, implementation determinants will likely differ in other regions of the world such as Sub-Saharan Africa.

In intervention research, there is growing attention to the concepts of outcome proximity (whether outcomes mirror intervention targets or skills in domains directly targeted by the intervention) and boundedness (whether an outcome is measured in a context that differs from the intervention context) [9]. In caregiver-mediated interventions, the vast majority of studies utilize behavioural coding on study-specific scales of intervention-specific skills. The limitation of this approach is that these types of measures may only identify transient and limited changes. A recommended approach to assess clinically meaningful child gains related to the intervention, is to use measures that do not only detect changes in intervention targets (i.e., caregiver strategies taught during coaching) but capture clinically meaningful change, and measures that are administered in a context different from the intervention (i.e., child’s skills assessed during interaction with a clinician vs. during a caregiver-child interaction) in order to assess generalization of child skills across different interaction partners.

### Proof-of-principle for cascaded task-sharing intervention in South Africa

Over the past eight years, the Center for Autism Research in Africa at the University of Cape Town has been studying various feasible approaches to early autism intervention ([14], p. 99–132). Our specific programme of research utilizes the Community-Early Start Denver Model (C-ESDM) materials. C-ESDM materials are open access, web-based, and designed to support families in low-resource contexts to learn NDBI strategies [34, 35]. C-ESDM modules include strategies to: (i) increase child attention to people; (ii) increase child communication; (iii) create joint activity routines; and (iv) improve caregiver understanding of antecedents, behaviours, and consequence, to help teach new behaviours.

**Figure 1:**
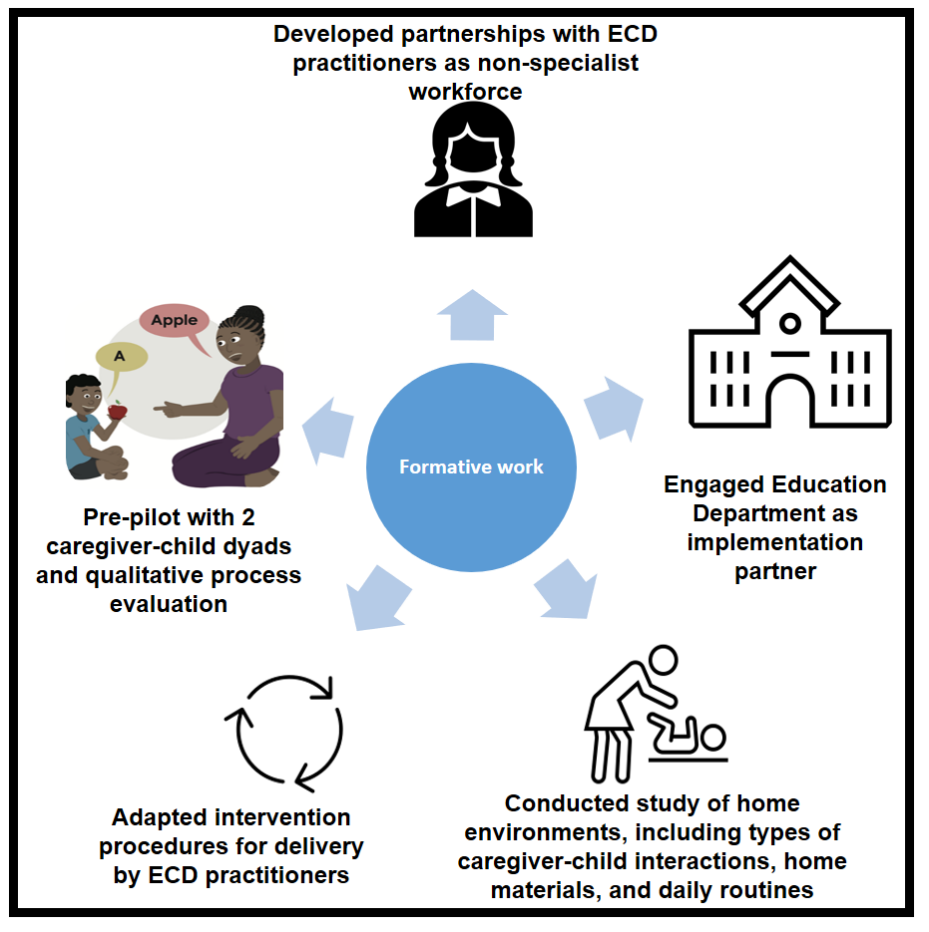
Formative activities that informed proof-of-principle cascaded task-sharing NDBI.

In the formative stages of our work we conducted five activities that set us up to complete the proof-of-principle pilot study of a cascaded task-sharing intervention in South Africa described in this manuscript (See Fig. 1).

**Figure 2:**
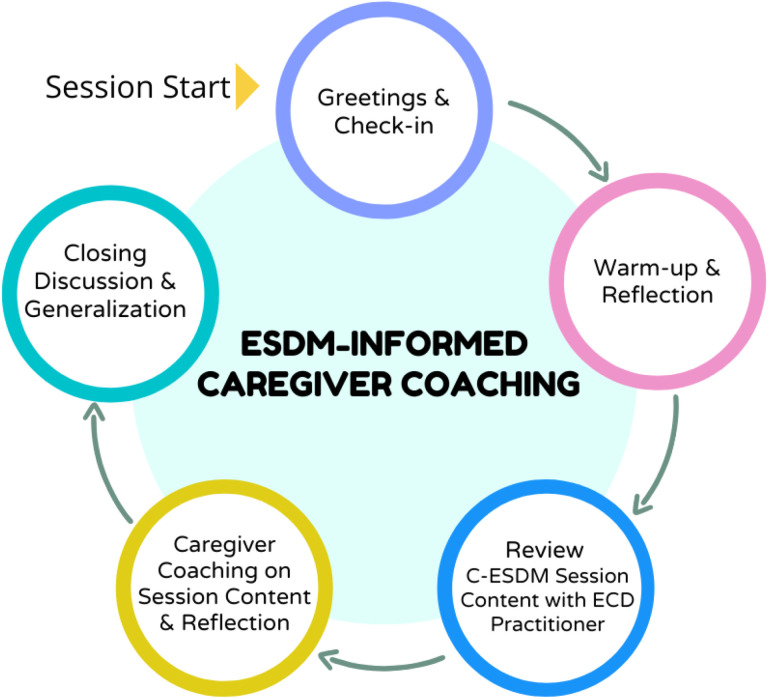
Caregiver coaching session structure of the proof-of-principle cascaded task-sharing NDBI.

First, we identified ECD practitioners, a non-specialist workforce supported by National policy and employed by the Western Cape Education Department, as non-specialist providers who could coach caregivers in NDBI strategies [18]. Second, we identified the Western Cape Education Department as an implementation partner [18]. Importantly, the Education Department oversees the ECD workforce, an alignment of non-specialist workforce and system of care which could support future scale-up efforts [36]. Coaching sessions were conducted at schools, with children on waiting lists for special education services, meaning participants were identified as autistic by the Western Cape Education Department but were not yet enrolled in school due to limited capacity to meet demand. Third, we identified caregiver preferences for early intervention, and examined whether joint activity routines, in which intervention strategies can be embedded, were applicable in low-resource, culturally diverse contexts in South Africa [30, 37]. Fourth, we adapted the training approach and session structure for non-specialist delivery. Modifications were made by an ESDM certified trainer and South African ESDM certified therapists, who were familiar both with intervention strategies and the South African context. A 4-day in-person training, led by South African ESDM therapists, was attended by ECD practitioners and their direct school supervisors. During the training C-ESDM provider materials introduced caregiver coaching concepts and core NDBI strategies, and ECD practitioners worked with a caregiver-child dyad to practice these strategies. The apprenticeship model for lay counsellor supervision in mental health informed ongoing ECD practitioner supervision [38]. Session structures for 12, one-hour, coaching sessions were created by the research team (See Fig. 2). A new intervention strategy was introduced with C-ESDM materials in each coaching session. The ECD practitioner then coached the caregiver in the new session skill across at least 2 caregiver-child activities. After each coaching activity, the ECD practitioner supported caregiver reflection. The session concluded with a discussion of the session skill and caregiver thoughts on how to practice the new skill across various caregiver-child routines.

Fifth, we conducted a pre-pilot study with 2 caregiver-child dyads and completed a qualitative process evaluation of the adapted coaching approach which identified preliminary implementation determinants (barriers and facilitators) [39]. Efforts were then made to capitalize on facilitators and to mitigate barriers. Further adaptations to the intervention approach included: (i) creating and displaying simple visual aids during each coaching session to focus ECD practitioners and caregivers on session key points, (ii) focusing ongoing ECD practitioner supervision on specific coaching behaviours, including being ‘collaborative’ and ‘reflective’, (iii) school partners allocating coaching space and a laptop for sessions, and protecting ECD practitioner time to conduct sessions, (iv) downloading of C-ESDM modules onto school computers, given limited access to reliable internet (with the permission of intervention developers), and (v) building in flexibility into the coaching schedule to account for caregiver public transportation delays.

Building on our formative work, in this proof-of-principle pilot study, we set out to answer two sets of critical questions about our cascaded task-sharing NDBI approach that required exploration. First, we wanted to determine whether our approach impacted fidelity of implementation of both non-specialist coaches and caregivers. Specifically, whether coaching by non-specialist providers resulted in improvements in caregiver use of intervention strategies with their young autistic child and whether coaches were able to adhere to coaching procedures as outlined in fidelity checklists. Second, we wanted to assess whether coaching impacted key short-term child and caregiver outcomes. Specifically, whether signals of change were detected in child social and communication abilities and caregiver stress and sense of competence. These questions were important to answer as without information on implementation fidelity and potential child and caregiver impact, larger-scale clinical trials may not be warranted.

## Methods

### Study Design

This proof-of-principle pilot study utilized a single-arm pre-post design to evaluate the fidelity of implementation by coaches (non-specialist ECD practitioners) and caregivers, and to explore short-term caregiver and child outcomes of NDBI-informed caregiver coaching by non-specialist ECD practitioners employed by the Western Cape Education Department in Cape Town, South Africa.

### Participant characteristics

#### Caregiver-Child Dyads:

Partner schools in the Western Cape Education Department identified children who were on their waiting list for autism special education services to participate in the pilot study. Inclusion criteria for caregiver-child dyads were as follows: i) child met DSM-5 criteria for autism spectrum disorder (ASD) [40], which was informed by a clinical evaluation that included an Autism Diagnostic Observation Schedule, second edition (ADOS-2; [41]) administered by research reliable clinicians, ii) the child was between the ages of 18–72 months, iii) primary family language was isiXhosa, isiZulu, Afrikaans, or English, iv) participant self-declared race was Black or Coloured (a South African term for mixed-race), and v) the caregiver was 18 years or older. Exclusion criteria for caregiver-child dyads were as follows: child with significant sensory or motor impairments, major physical abnormalities, presence of a neurological disorder of known etiology (e.g., Fragile X syndrome), history of serious head injury and/or neurological disease, or caregiver-child dyads that were unable to attend assessments and the 12 coaching sessions.

#### ECD Practitioners:

Autism schools in the Western Cape Education Department identified ECD practitioners to participate in caregiver coaching [18]. Inclusion criteria for ECD practitioners were: i) employed by the Western Cape Education Department Schools, and ii) involved in delivery of caregiver coaching sessions.

### Measures

#### Fidelity Scales

The ESDM Caregiver Fidelity Scale provides a method for assessing the fidelity with which a caregiver uses ESDM strategies in a joint activity routine with their young child [42]. The 13-item rating scale includes ratings of performance from 1 to 5. The ESDM Coaching Fidelity Scale evaluates 13 coaching behaviours during the caregiver coaching sessions [42]. Items are individually rated with a Likert-type rating scale with values that vary from 1 to 4, with higher scores reflecting a greater degree of fidelity. A portion of the items evaluate specific activities within the intervention session (e.g., warm-up activity, coaching on the topic) and the remaining items assess coaching characteristics evaluated across the entire session (e.g., reflective, non-judgmental). The ESDM Coaching Fidelity Rating and the P-ESDM Caregiver Fidelity was completed through consensus coding of video-recorded sessions by the research team.

##### Child Development:

Griffiths Scales of Child Development, Third Edition (Griffiths-III; [43]) is a comprehensive developmental assessment designed to evaluate children ranging in ages from birth to 5 years 11 months. The Griffiths-III provides a profile of both strengths and weaknesses in child development across 5 domains: foundations of learning, language and communication (expressive/receptive language), eye and hand coordination (fine motor skills and visual perception), personal-social-emotional (emotional development and social interactions), and gross motor (postural control, balance, and body coordination). Developmental quotients (DQs) were calculated as developmental age/chronological age. While the Griffiths is not standardized in South Africa, this assessment is widely used by clinicians in South Africa and South African researchers advised on restructuring certain items on the latest version in order to make the assessment more culturally fair [44]. Furthermore, the construct validity of the Griffiths was evaluated in a study of 430 South African children from four racial groups (white, mixed race, Asian and black) and results demonstrated that patterns of correlation for South African and British participants (standardization sample) were similar suggesting that the Griffiths measures a construct that is consistent across cultures [44].

#### Child Adaptive Behaviour

The Vineland Adaptive Behaviour Scales, Third Edition (VABS-3; [45]) is a measure of adaptive functioning in individuals ranging in age from birth to 90. The VABS-3 was designed to assess adaptive behaviour in the domains of socialization (play, interpersonal relationships and coping skills), communication (receptive, expressive and written language skills), daily living skills (personal, domestic, and community living skills), and motor skills (gross and fine motor). The caregiver-report form is designed to gather information from adult (caregiver) respondents who are knowledgeable about the every-day functioning of their child, and item responses are collected on a 3-point Likert scale with values representing 0 (never), 1 (sometimes), and 2 (usually or often) to capture the frequency of each target behaviour.

#### Caregiver Sense of Competence

The Parent Sense of Competence Scale (PSOC; 46) is a 16-item self-report questionnaire designed to measure the degree to which caregivers feel competent and confident in ‘parenting’ their children (i.e., efficacy) and the quality of affect associated with ‘parenting’ (i.e., satisfaction). Items are rated on a 6-point Likert scale with high scores representing high degrees of satisfaction and efficacy. The Satisfaction subscale reflects ‘parenting’ frustration, anxiety, and motivation, whereas the Efficacy subscale assesses capability, problem-solving ability, and competence.

##### Caregiver Stress:

The Parenting Stress Index-Short Form (PSI-SF; [47]) is a 36-item self-report questionnaire that is designed to measure ‘parenting’ stress. Items on the PSI-SF are rated on a 5-point Likert scale ranging from 1 (strongly agree) to 5 (strongly disagree) with three reverse-scoring items. It includes three subscales with 12 items each: parenting distress, parent–child dysfunctional interaction, and difficult child. The PSI-SF score is an indicator of parenting stress associated with parental anxiety, interactions with their children and child behaviours. Higher scores relate to higher parenting stress.

### Data Analysis

Participant characteristics and summary statistics (*n, percentage for categorical variables and range, median and inter-quartile range* (*IQR*) for continuous and score variables) were computed separately for each participant group (i.e. caregiver, child, and ECD practitioner groups). Due to the limited sample size, pre-to-post summary statistics (median and IQR) were presented alongside participant trajectories to visually display individual-level data. Comparison of both group and individual change from baseline (T1) to follow-up assessment (T2) are visualized using box plots and connected dot-plots, respectively. Analysis was performed using R (Version 4.1.1; [48]). Given the exploratory nature of group comparison with a small sample size, the non-parametric Wilcoxon signed rank test for paired samples was used to compare group medians of the following scores between the baseline (T1) and post (T2) intervention time points: ECD practitioner (ESDM Coaching Fidelity Scale) scores, child (Vineland and Griffiths), and caregiver (ESDM Caregiver Fidelity Scale, Caregiver Stress and Sense of Competency) scores. Reporting of results is in accordance with the CONSORT statement for pilot and feasibility trials [49].

### Ethics, Protocol Registration and Funding

Given that this study was a collaboration between the Center for Autism Research in Africa at the University of Cape Town (UCT) and the Duke Centre for Autism and Brain Development at Duke University all study procedures were submitted and approved by both ethical review boards (UCT: HREC 301/2015 and 468/2019; Duke: IRB Pro00103045 and Pro00064533). Study procedures were reviewed with participants and informed consent was collected prior to data collection. The study protocol was registered on ClinicalTrials.gov (NCT04068688). Study funding provided by the National Institute of Mental Health and the Fogarty International Center (R21 MH120696). As the study was funded by the National Institute of Mental Health all data has been submitted to ClinicalTrials.gov and the National Database for Autism Resarch (NDAR).

## Results

### Participant flow

**Figure 3:**
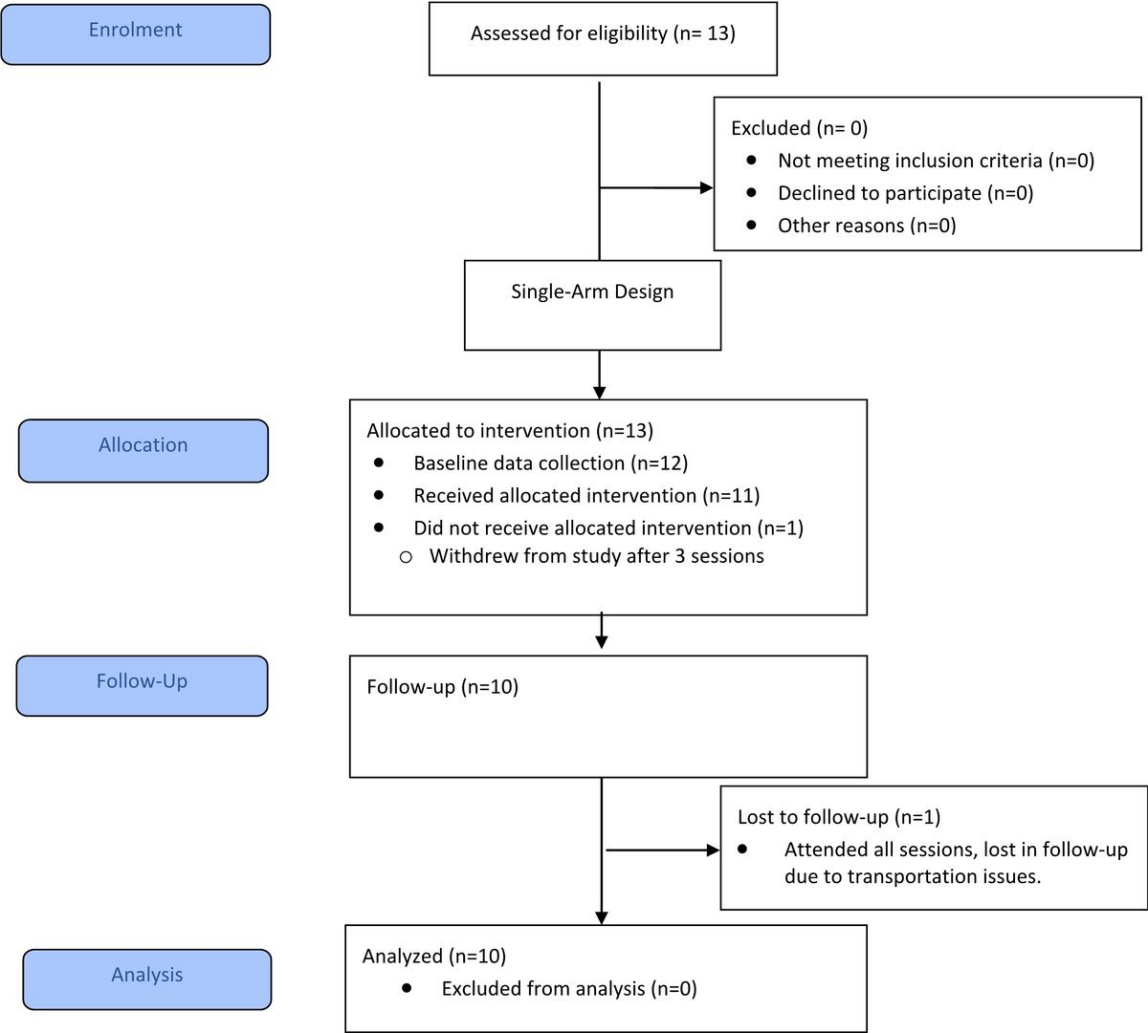
Consort Flow Diagram

The CONSORT flow chart in Fig. 3 shows the number of participants invited to participate, numbers that consented, enrolled, received intervention, and/or withdrew. Thirteen caregiver-child dyads were invited to participate and consented. One caregiver-child dyad withdrew from the study prior to the start of the intervention (due to travel difficulties), and 1 caregiver-child dyad withdrew after 3 coaching sessions (due to time constraints). Ten caregiver-child dyads completed all baseline and follow-up assessments and attended all 12 coaching sessions. One caregiver-child dyad was lost to follow-up, but received all 12, 1-hour coaching sessions. Data were collected between April 2018 and November 2019. Intervention took place over an average of 109 days (range: 91–135). Participants completed baseline testing an average of 24 days (range: 10–46) prior to Session 1. After session 12, follow-up evaluation was completed by participants on average 15 days (range: 1–61) after their final session.

### Participant Characteristics

Child and caregiver baseline characteristics are outlined in Table 1. In terms of child characteristics (n = 12) the median child age was 52 months (range 35–64) and 11 were male (caregiver reported child gender). The median age of autism diagnosis was 38 months (range 24–48). As part of diagnostic confirmation an ADOS-2 module 1 was completed with 11 children (median comparison score 7, range 4–9). In terms of baseline caregiver characteristics (n = 12) the median caregiver age was 36 years (range 29–60) of which 6 caregivers were mothers, 4 were fathers, and 2 were grandmothers. In terms of caregiver education 6 had attained grade 12 or less, and 6 had a post-graduate diploma or tertiary education. Nine were married, 8 were unemployed, and 7 reported a monthly household income between R4,501-R12,500 (~$290-$806). In terms of perceived economic security seven reported that they were “just getting by” or “struggling” and 6 were dependent on public transportation.

The non-specialist ECD practitioner group (n = 4) consisted of three bilingual (English and Afrikaans) Coloured females, and one trilingual (English, Afrikaans and isiXhosa) Black female. One of the ECD practitioners had a Grade 12 education (completed secondary education) and the other three had post-Grade 12 Certificates.

### Fidelity of Caregiver Implementation and ECD Practitioner Coaching

**Figure 4:**
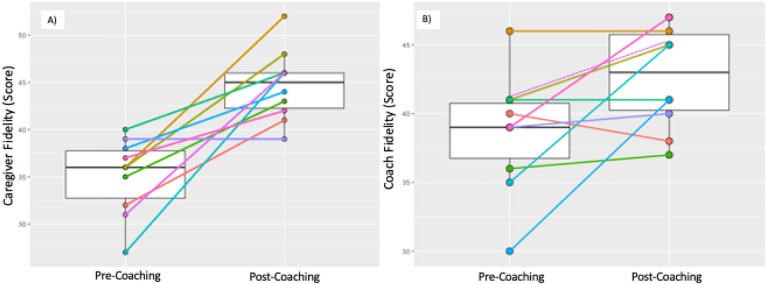
Box plots of Pre- and Post-ESDM Caregiver and Coach fidelity: median, IQR, and Pre- and Post-individual Caregiver and Coach fidelity trajectories.

We examined individual and group change from baseline (T1) to follow-up assessment (T2) in implementation fidelity of caregivers (n = 10) and ECD practitioners (n = 4). Results are shown in Table 2 and in Fig. 4. Caregiver implementation fidelity increased significantly from T1 to T2 (median (IQR) T1 = 36 (32,38); T2 = 45 (42,46); *p* = 0.009). Notably, fidelity of implementation improved in 9 out of 10 caregivers and remained the same for the 10th caregiver (see Fig. 4A). Similarly, non-specialist ECD practitioners demonstrated a significant increase in coaching fidelity between T1 and T2 (median (IQR) T1 = 39 (36,41); T2 = 43 (40,46); *p* = 0.042). At an individual level, coaching fidelity increased in 7/10 dyads coached, remained the same with 2 dyads and dropped in one (see Fig. 4B).

### Short-term Outcomes: Children

**Figure 5:**
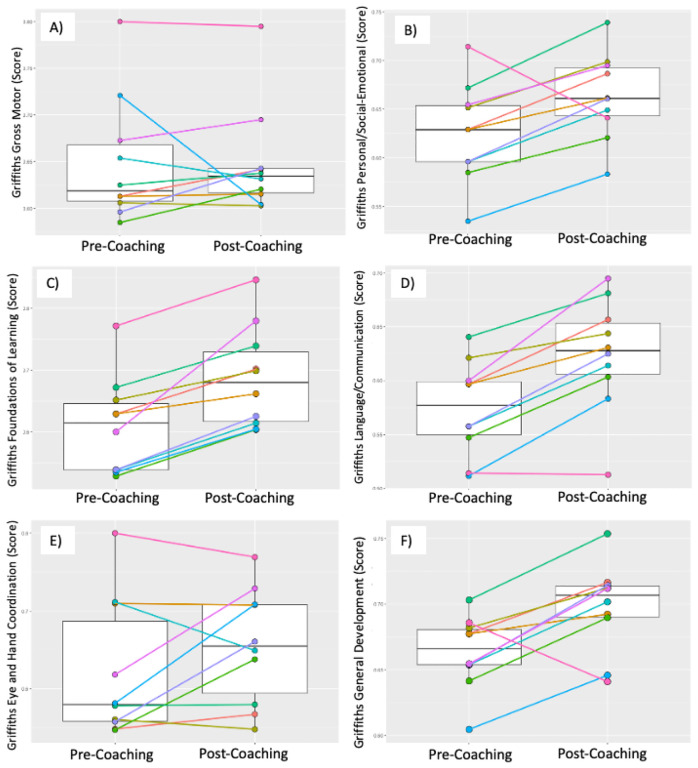
Box plots of Pre- and Post-Griffiths Scales of Mental Development-III (Griffiths-III): A: Gross Motor; B: Personal/Social Communication; C: Foundations of Learning; D: Language & Communication; E: Eye-Hand Coordination; F: General Development DQs with median and IQR; and Pre- and Post-individual Griffiths-III DQ trajectories.

**Figure 6:**
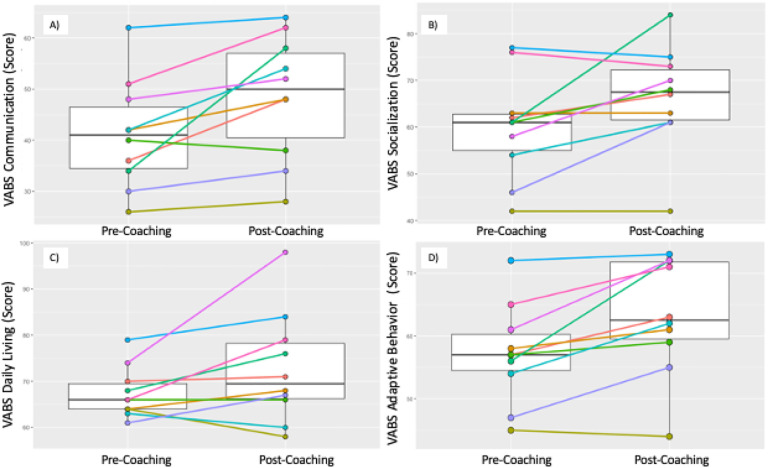
Box plots of Pre- and Post-Vineland Adaptive Behaviour Scales, Third Edition (VABS-3): A: Communication; B: Socialization; C: Daily Living Skills; D: Adaptive Behaviour Subscale Standard Scores with median and IQR; and Pre- and Post-individual VABS-3 Subscale Standard Score trajectories.

Given the small sample size and ‘proof-of-principle’ nature of the study, we evaluated group and individual change from T1 to T2 on the pre-selected outcome measures - Griffiths-III and VABS 3. Results are shown in Table 2 and in Figs. 5 and 6. On the Griffiths-III Gross Motor DQ no significant increase was observed between T1 and T2 (median (IQR) T1 = 0.62 (0.61,0.67); T2 = 0.63 (0.62,0.64); *p* = 0.557). Griffiths-III Gross Motor DQ improved in 5 out of 10 children and dropped in 5 (see Fig. 5A). While the Griffiths-III Personal/Social-Emotional DQ also did not show a significant group-based increase (median (IQR) T1 = 0.63 (0.60,0.65); T2 = 0.66 (0.64,0.69); *p* = 0.084), the Personal/Social-Emotional DQ improved in 8 out of 10 children, and dropped in 2 (see Fig. 5B). The Griffiths-III Foundations of Learning DQ showed a significant group-based increase (median (IQR) T1 = 0.61 (0.54,0.65); T2 = 0.68 (0.61,0.74); *p* = 0.002), with all 10 children demonstrating improved Foundations of Learning DQ scores (see Fig. 5C). The Griffiths-III Language/Communication DQ also showed a significant group-based increase (median (IQR) T1 = 0.58 (0.55,0.60); T2 = 0.63 (0.60,0.66); *p* = 0.004). Language/Communication DQ improved in 9 out of 10 children and dropped in one (see Fig. 5D). Griffiths-III Eye and Hand Coordination DQ did not show a significant increase group-based increase (median (IQR) T1 = 0.58 (0.56,0.71); T2 = 0.65 (0.58,0.71); *p* = 0.27). Eye and Hand Coordination DQ improved in 6 out of 10 children, 2 stayed the same, and 2 dropped (see Fig. 5E). Finally, Griffiths-III General Development DQ showed a significant group-based increase (median (IQR) T1 = 0.67 (0.65,0.68); T2 = 0.71 (0.69,0.71); *p* = 0.02). General Development DQ improved in 9 out of 10 children and dropped in 1 child (see Fig. 5F).

The VABS-3 Communication Standard Scores increased significantly between T1 and T2 (median (IQR) T1 = 41 (34,48); T2 = 50 (38,58); *p* = 0.011). Notably, VABS-3 Communication Standard Scores improved in 9 out of 10 children and dropped in one (see Fig. 6A). VABS-3 Socialization Standard Scores also showed a significant group-based increase (median (IQR) T1 = 61 (54,63); T2 = 67.5 (61,73); *p* = 0.042). VABS-3 Socialization Standard Scores improved in 6 out of 10 children, 2 stayed the same, and 2 dropped (see Fig. 6B). VABS-3 Daily Living Standard Scores did not show a significant group-based increase (median (IQR) T1 = 66 (64,70); T2 = 69.5 (66,79); *p* = 0.086). VABS-3 Daily Living Standard Scores improved in 7 out of 10 children, stayed the same in 1, and dropped in 2 (see Fig. 6C). Finally, on the VABS-3 Adaptive Behaviour Standard Score a significant increase was observed (median (IQR) T1 = 57 (54,61); T2 = 62.5 (59,72); *p* = 0.009). VABS-3 Adaptive Behaviour Standard Score improved in 9 out of 10 children and dropped in 1 (see Fig. 6D).

### Short-term Outcomes: Caregivers

**Figure 7:**
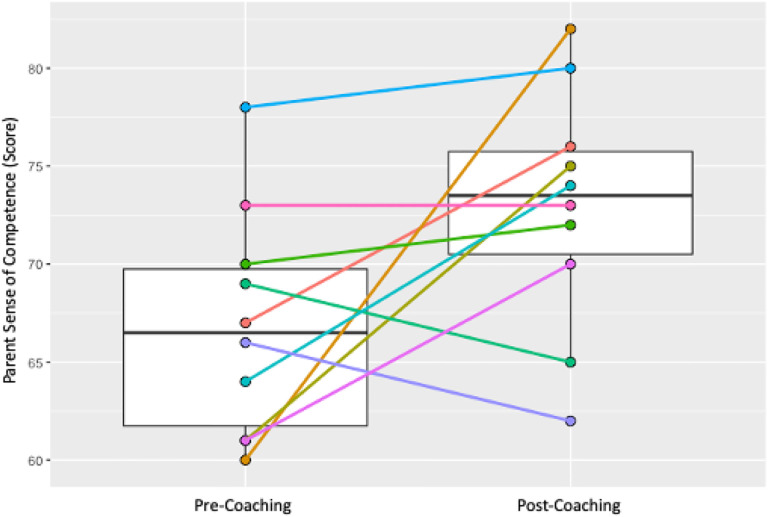
Box plots of Pre- and Post- Caregiver Sense of Competence Total Scores: median, IQR, and Pre- and Post-individual Caregiver Sense of Competence Total Scoretrajectories.

**Figure 8:**
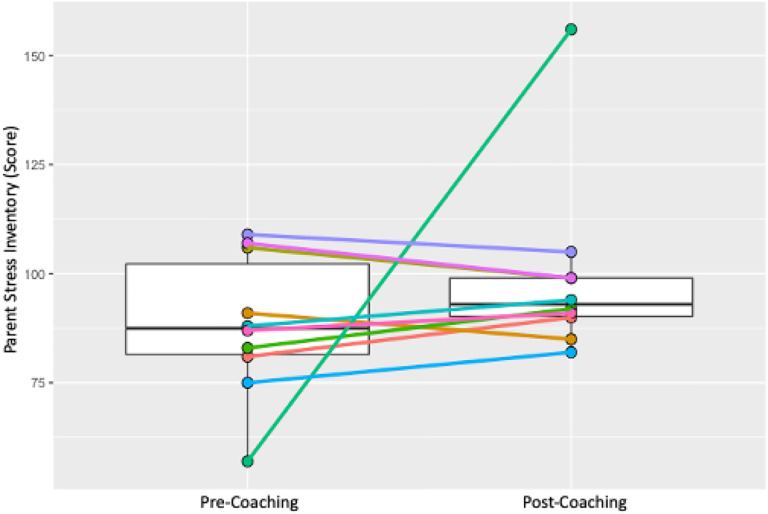
Box plots of Pre- and Post- Parenting Stress Index Total Score: median, IQR; and Pre- and Post-individual Parenting Stress Index Total Score trajectories.

Individual and group changes were measured across two pre-specified caregiver outcomes – caregiver sense of competence and caregiver stress. Results are shown in Table 2 and in Figs. 7 and 8. Caregiver sense of competence did not show a significant group-based increase (median (IQR) T1 = 66.5 (61,70); T2 = 73.5 (70,76); *p* = 0.075). Sense of competence total scores improved in 7 out of 10 caregivers and dropped in 3 (see Fig. 7). Caregiver stress total scores did not show a significant group-based decrease (median (IQR) T1 = 87.5 (81,106); T2 = 93 (90,99); *p* = 0.332). Caregiver stress total scores increased in 6 out of 10 caregivers and dropped in 4 (see Fig. 8).

## Discussion

In this proof-of-principle study of a 12-session low-intensity cascaded task-sharing NDBI delivered by non-specialist caregiver coaches, we utilized a single-arm pre-post design and evaluated fidelity of caregivers and non-specialist coaches to programme delivery, as well as targeted short-term child and caregiver outcomes. The 12, 1-hour coaching sessions were delivered over an average of 115 days (~ 4 months). The coaching was conducted by Early Childhood Development (ECD) practitioners in public schools in Cape Town, South Africa. Caregiver-child dyads were identified by partner schools, from their waitlist of young autistic children who were eligible for services, but not enrolled in in-person instruction. To our knowledge, this is the first study to evaluate a cascaded task-sharing NDBI caregiver coaching approach delivered by non-specialist ECD practitioners in any Sub-Saharan African country.

All caregivers demonstrated improvements in fidelity scores across coaching sessions which suggests that non-specialist ECD practitioners were able to support growth in caregiver implementation of NDBI strategies. Increases in ECD practitioner coaching fidelity was also documented across coaching sessions which suggests that non-specialists increased their competency in coaching behaviours. Of note, ECD practitioners had only completed a 4-day in-person training prior to starting caregiver coaching sessions – and were otherwise naive to both the coaching approach and NDBI strategies.

Given the sample size and proof-of-principle nature of the study, results reported are exploratory in nature but were included to determine if key outcome measures changed in the desired direction and to identify outcome measures that may be more or less sensitive to change in a non-specialist led task-sharing intervention. The study was therefore ‘signal-seeking’ in terms of outcome measures. It was very encouraging that not only was growth in caregiver-implementation fidelity observed (measured using a behavioural coding approach on study-specific scales of intervention-specific skills), but significant growth was also seen on two Griffiths-III subscales (Language/ Communication and Foundations of Learning) and on the General Development DQ. Significant growth was also seen on and two VABS-3 subscales (Communication and Socialization) and the Adaptive Behaviour Standard Score. The Griffiths-III and the VABS-3 assess developmental and adaptive domains, which are not direct intervention targets and are measures that are administered in contexts different from the intervention suggesting generalization of child skills across interaction partners.

While not statistically significant, an increase in caregiver sense of competence was observed in 7 out of 10 caregivers, suggesting increases in confidence in ‘parenting’ and ‘parenting’ satisfaction. In addition, although not statistically significant, an increase in total stress composite scores on the PSI-SF post-intervention were noted, suggesting increases in ‘parenting’ stress. However, given the sample size, this increase in ‘parenting’ stress appeared to be mostly driven by a large increase in a single participant’s score.

Perhaps a unique characteristic of this study was the profile of caregivers who completed coaching. Given primary caregivers critical role in caregiver-mediated interventions, and the transactional relationship of caregiver-coaching whereby caregivers can *both* be impacted by the intervention and impact intervention outcomes, caregiver characteristics are particularly relevant to track [27]. Most caregivers represented in autism intervention research are white female (mothers) from upper-middle income families in high-income, English-speaking countries [50]. In this pilot, more fathers and grandmothers completed coaching than mothers. This is important because the impact of coaching on fathers’ and grandmothers’ coping skills, stress management, and self-efficacy is under-researched particularly in culturally and linguistically diverse groups and may differ from responses of mothers. In societies where caregiving roles are more broadly defined, such as in sub-Saharan Africa, contextual understanding of responses to autism caregiver coaching across caregiver type will be important to understand [51–52]. Furthermore, contextual factors that threaten family adaptive functioning, such as living in poverty, are prevalent in low-resource contexts like South Africa [28–31]. All caregivers in this study were Black or Coloured (a South African term for mixed-race), groups that are significantly impacted by poverty and structural inequalities in South Africa [17] At baseline 8 caregivers reported being unemployed, and 7 reported that they were struggling or just getting by financially. Given the sample size and proof-of-principle nature of the study, examination of the degree to which response to intervention was moderated by caregiver-characteristics was not possible, but would be important to consider in the design and implementation of caregiver-mediated intervention studies in this context going forward.

### Limitations

First, we acknowledge the importance of a mixed-method (i.e. a combined qualitative-quantitative) intervention evaluation approach to provide a more comprehensive and nuanced understanding of research in novel contexts. The absence of any qualitative data to inform interpretation of these results was therefore a limitation of this study. We had in fact planned a qualitative component. However, this study was unexpectedly interrupted by the COVID-19 pandemic, which resulted in all study activities being suspended then pivoting to telehealth, and we were therefore not granted permission to complete qualitative interviews in a timely fashion [53]. However, the approach used in this proof-of-principle study was informed by extensive qualitative formative research with multiple stakeholder groups in addition to a qualitative process evaluation [30, 37, 39, 54]. Second, we also acknowledge that key outcomes measures utilized in this study (i.e. Griffiths-III, VABS-3) have not yet been formally validated in South Africa. Lack of access to validated tools remains a significant barrier to autism research in low-resource contexts globally [55]. The Griffiths-III and VABS-3 were chosen because they are widely used by clinicians in South Africa, suggesting a degree of face validity. While not formally validated, the construct validity of the Griffiths has been evaluated in South Africa, and results suggest that measured constructs are consistent across cultures [44], and the VABS has been evaluated in a South African PhD thesis with results suggesting that the instrument was ‘useful and valid’ for people up to 22 years of age [56]. Assessments were also completed by multilingual clinicians (clinical psychologists at the PhD/Masters level), who were trained to reliability. A third limitation of our work is that assessors and caregivers were not masked to intervention status, as this study utilized a single-arm, pre-post design. It is therefore important to underline that this proof-of-principle study cannot make claims about intervention efficacy due to study design, sample size, and lack of masking. However, this is the first study in Africa to evaluate a cascaded task-sharing approach, and we therefore aimed to understand both coach and caregiver implementation fidelity alongside short-term outcomes in order to set the stage for a future intervention study designed to assess efficacy. Finally, C-ESDM video content utilized in the caregiver-coaching sessions were US-based and mismatched for the South African context [39]. These materials were used both in the 4-day training of the ECD practitioners and to introduce NDBI strategies in each coaching session. Formal adaptation of the intervention materials, to closely match the South African context is an important next step.

### The potential relevance of cascaded task-sharing NDBI to other low-resource contexts and future directions

While limited by sample size and study design, the questions asked and answered in this proof-of-principle study are important as without information on caregiver and non-specialist coach implementation fidelity and potential child and caregiver impact, larger-scale clinical trials may not be warranted. Task-sharing caregiver coaching to non-specialist providers, who are integrated into existing systems of care, is a key implementation strategy that may support scale-up. Such an approach may be well suited to diverse, low-resource settings with limited supports and services for young autistic children. This approach will likely gain traction over time as stretched systems of care in low resource countries are tasked with supporting not only the physical health but also the developmental needs of all children, including those with developmental disabilities [57]. As noted, the need for innovative solutions is particularly evident on the African continent, which is undergoing an unprecedented demographic shift in child population size.

Now that there is proof-of-principle, larger scale studies are needed to expand on the evidence-base and answer the next set of key questions. It will be important that these studies include the perspectives of diverse stakeholders, including those with lived experience, in their design and implementation. This is an important way to decrease the research-to-practice gap and ensure that end-products are acceptable across stakeholder groups [58]. Moving forward it will be key to understand the following: First, whether a scalable, non-specialist delivered, low-intensity caregiver coaching intervention for young autistic children that is contextually adapted for the South African context and integrated into an existing system of care, can significantly improve both clinically meaningful short-term and long-term child and caregiver outcomes. Second, whether this is a cost-effective approach. This is a key question for policy and decision makers who would need to adopt and support scale-up. Third, what the key determinants (barriers and facilitators) that impact successful implementation of the cascaded task-sharing NDBI approach in South Africa are. Understanding implementation barriers, ways to mitigate their impact, and implementation facilitators, will inform the development of specific implementation strategies that would support scale-up.

## Conclusion

This was the first study, to our knowledge, to evaluate a non-specialist delivered cascaded caregiver coaching of an NDBI for autism in Sub-Saharan Africa. This study provides a novel contribution to the literature by exploring a potentially scalable coaching intervention in Africa, utilizing a cascaded task-sharing approach delivered within an existing system of care. In keeping with the proposals set out in the *Lancet* Commission for future care and clinical research in autism, this study is a small but critical step toward offering feasible and accessible services and supports, embedded within communities, drawing on local expertise to address the diverse needs of families [7] This study is timely given growing recognition of the importance of early autism intervention, emerging policies in LMIC that provide a framework on which to build intervention services, and global programmes that support child developmental needs [1, 15, 59].

## Figures and Tables

**Table 1:** Participant Characteristics at Baseline (Caregivers and Children) for caregiver-child dyads (n = 12)

*Child Characteristics*	Median (IQR)^a^	Range
**Child Age in months**	52 (48.75, 61)	35–64
**Male, n (%)**	11 (91.7)	-
**Female, n (%)**	1 (8.3)	-
**Age of first developmental concern in months**	24 (24, 36)	17–40
**Age of diagnosis in months (ASD)**	38 (30, 46)	24–48
**ADOS-2 module completed, n (%)**		
Module 1	11 (91.7)	-
Module 2	1 (8.3)	-
**ADOS-2 comparison score**		
Module 1	7 (6, 7.5)	4–9
Module 2	6 (-,-)*	-
Caregiver Characteristics	N (%)^a^	Range
**Caregiver age in years**, M (SD)	36 (34, 37.5)	29–60
**Number of siblings living in the home**		
No siblings	2 (16.7)	-
1 sibling	5 (41.7)	-
2 siblings	4 (33.3)	-
3 + siblings	1 (8.3)	-
**Primary Caregiver**		
Mother	6 (50))	-
Father	4 (33.3)	-
Grandmother	2 (16.7)	-
**Caregiver Education**		
< Grade 9	1 (8.3)	-
Grade 12	5 (42)	-
Post-Grade 12 Diploma/Certificate	3 (25)	-
Tertiary	3 (25)	-
**Marital Status**		
Married	9 (75)	-
Live-in Partner	2 (16.7)	-
Single	1 (8.3)	-
**Employment Status**		
Not working	8 (67)	-
Working Part-time	1 (8)	-
Working Full-time	3 (25)	-
**Household Income/month**		
R4,501–R12,500 (~$290–$806)	7 (58.3)	-
R12,501–R30,000 (~$>806–$1,935)	5 (41.7)	-
**Perceived economic security**		
Struggling	1 (8.3)	-
Just getting by	6 (50)	-
Doing ok	5 (41.7)	-
Managing well	0	-
Well off	0	-
**Reliance on public transport, n (%)**	6 (50)	-

a Unless otherwise noted;

* Only one person completed ADOS-2, Module 2

**Table 2:** Median and IQR of caregiver fidelity, coach fidelity, and caregiver and child outcomes.

	Time 1		Time 2	
	Median	IQR	Median	IQR
***Fidelity Scales***				
ESDM Caregiver Fidelity*	36	32,38	45	42,46
ESDM Coaching Fidelity*	39	36,41	43	40,46
Child Outcomes				
**VABS-3**				
Communication*	41.00	34.00,48.00	50.00	38.00,58.00
Socialization*	61.00	54.00,63.00	67.50	61.00,73.00
Daily Living Skills	66.00	64.00,70.00	69.50	66.00,79.00
Adaptive Behaviour*	57.00	54.00,61.00	62.50	59.00,72.00
**Griffith’s III DQ**				
Gross Motor	0.62	0.61,0.67	0.63	0.62,0.64
Personal, Social Emotional	0.63	0.60,0.65	0.66	0.64,0.69
Foundations of Learning*	0.61	0.54,0.65	0.68	0.61,0.74
Language | Communication*	0.58	0.55,0.60	0.63	0.60,0.66
Eye and Hand Coordination	0.58	0.56,0.71	0.65	0.58,0.71
General Development*	0.67	0.65,0.68	0.71	0.69,0.71
Caregiver Outcomes				
Parent Sense of Competency	66.50	61,70	73.50	70,76
Parent Stress Index	87.50	81,106	93.00	90,99

Griffiths III developmental quotient (DQ) = (developmental age/chronological age)

VABS-3: Vineland Adaptive Behaviour Scales, Third Edition

* Indicates statistically significant (p < 0.05) changes between T1 and T2

## Data Availability

As the study is NIH-funded all data has been submitted to ClinicalTrials.gov and the National Database for Autism Resarch (NDAR)

## Abbreviations

ASD: Autism Spectrum Disorder
ADOS-2: Autism Diagnostic Observation Scale (2^nd^ Ed.)
CESDM: Community-Early Start Denver Model
DSM5: Diagnostic and Statistical Manual (5^th^ Ed.)
NBDI: Naturalistic Developmental Behavioural Intervention
DQ: Developmental Quotient
ECD: Early Childhood Development
IQR: Inter-Quartile Range
LMIC: Low- and Middle-Income Country
PS-SF: Parenting Stress Index-Short Form
PSOC: Parent Sense of Competence Scale
VABS3: Vineland Adaptive Behaviour Scales (3^rd^ Ed.)
WHO: World Health Organization
